# The facial nerve palsy and cortisone evaluation (FACE) study in children: protocol for a randomized, placebo-controlled, multicenter trial, in a *Borrelia burgdorferi* endemic area

**DOI:** 10.1186/s12887-021-02571-w

**Published:** 2021-05-04

**Authors:** Sofia Karlsson, Sigurdur Arnason, Nermin Hadziosmanovic, Åsa Laestadius, Malou Hultcrantz, Elin Marsk, Barbro H. Skogman

**Affiliations:** 1 Center for Clinical Research Dalarna - Uppsala University, Region Dalarna County, Falun, Sweden; 2 Department of Clinical Science, Intervention and Technology (CLINTEC), Karolinska Institutet, Stockholm, Sweden; 3 Department of Otorhinolaryngology, Region Dalarna County, Falun, Sweden; 4 Department of Pediatric Infectious Diseases, Astrid Lindgren’s Children’s Hospital, Karolinska University Hospital, Solna, Sweden; 5 Uppsala Clinical Research Centre, Uppsala University, Uppsala, Sweden; 6 Department of Pediatric Nephrology, Astrid Lindgren’s Children’s Hospital, Karolinska University Hospital, Solna, Sweden; 7 Department of Women and Child Health, Karolinska Institutet, Stockholm, Sweden; 8 Department of Otorhinolaryngology, Karolinska University Hospital, Stockholm, Sweden; 9 Department of Pediatrics, Region Dalarna County, Falun, Sweden; 10 Faculty of Medical and Health Sciences, Örebro Universitet, Örebro, Sweden

## Abstract

**Background:**

Children with acute peripheral facial nerve palsy cannot yet be recommended corticosteroid treatment based on evidence. Adults with idiopathic facial nerve palsy are treated with corticosteroids, according to guidelines resulting from a meta-analysis comprising two major randomized placebo-controlled trials. Corresponding trials in children are lacking. Furthermore, acute facial nerve palsy in childhood is frequently associated with Lyme neuroborreliosis, caused by the spirochete *Borrelia burgdorferi*. The efficacy and safety of corticosteroid treatment of acute facial nerve palsy associated with Lyme neuroborreliosis, has not yet been determined in prospective trials in children, nor in adults.

**Method:**

This randomized double-blind, placebo-controlled study will include a total of 500 Swedish children aged 1–17 years, presenting with acute facial nerve palsy of either idiopathic etiology or associated with Lyme neuroborreliosis. Inclusion is ongoing at 12 pediatric departments, all situated in *Borrelia burgdorferi* endemic areas. Participants are randomized into active treatment with prednisolone 1 mg/kg/day (maximum 50 mg/day) or placebo for oral intake once daily during 10 days without taper. Cases associated with Lyme neuroborreliosis are treated with antibiotics in addition to the study treatment. The House-Brackmann grading scale and the Sunnybrook facial grading system are used for physician-assessed evaluation of facial impairment at baseline, and at the 1- and 12-month follow-ups. Primary outcome is complete recovery, measured by House-Brackmann grading scale, at the 12-month follow-up. Child/parent-assessed questionnaires are used for evaluation of disease-specific quality of life and facial disability and its correlation to physician-assessed facial impairment will be evaluated. Furthermore, the study will evaluate factors of importance for predicting recovery, as well as the safety profile for short-term prednisolone treatment in children with acute facial nerve palsy.

**Discussion:**

This article presents the rationale, design and content of a protocol for a study that will determine the efficacy of corticosteroid treatment in children with acute facial nerve palsy of idiopathic etiology, or associated with Lyme neuroborreliosis. Future results will attribute to evidence-based treatment guidelines applicable also in *Borrelia burgdorferi* endemic areas.

**Trial registration:**

The study protocol was approved by the Swedish Medical Product Agency (EudraCT nr 2017–004187-35) and published at ClinicalTrials.gov (NCT03781700, initial release 12/14/2018).

## Background

Treatment strategies regarding children with acute peripheral facial nerve palsy (FNP) are potentially suboptimal. The benefit of corticosteroid treatment in this group of patients has been debated for years without consensus, and up to now evidence of its efficacy is lacking [1]. The rationale for further research on the subject matter will be outlined in this background.

Acute FNP is a condition with several different etiologies. Less frequent causes of the condition include otitis media, infection by varicella zoster virus, trauma, malignancy and hypertension [2]. In adults, the vast majority of cases are classified as idiopathic FNP, traditionally referred to as Bell’s palsy [3]. In children, Lyme neuroborreliosis (LNB) is the most common etiology of acute FNP in *Borrelia burgdorferi* endemic areas. In studies from Norway and Sweden, acute FNP in children was associated with LNB in 65 and 58% of cases respectively [4, 5]. The spirochete *Borrelia burgdorferi* sensu latu, causing LNB, is transmitted by ticks. Lyme disease is the most common vector-borne infection in temperate areas of the northern hemisphere and may give rise to a disseminated infection, advancing from the skin into the nervous system [6]. Among children with LNB, acute FNP is the most common specific neurological finding [7]. An acute FNP associated with LNB is treated with antibiotics (ceftriaxone or doxycycline) according to guidelines [8].

The incidence of acute FNP in childhood differs between studies, due to variations in settings and definitions of the condition. An extensive population-based study from Northern California, USA, shows an annual incidence of idiopathic FNP of 18.8/100000 children (≤18 years old) [9]. From a European *Borrelia burgdorferi* endemic area, a large Norwegian population-based study reported an incidence of 21/100000 per year of FNP of various etiologies among children [4]. In a Swedish study the corresponding number was 30/100000 children per year [5]. Whether or not the study setting represents an area endemic to *Borrelia burgdorferi,* would reasonably affect the incidence of acute FNP in the pediatric population. However, the local routine work-up regarding clinical evaluation of the condition (and hence the classification of LNB cases), may vary between and within countries resulting in difficulties when comparing data.

Acute FNP in children has previously been described as a benign condition with excellent outcome. Admittedly, the spontaneous course in children generally results in higher recovery rates than in adults [3]. However, some publications suggest that the condition is not as benign as supposed. Biebl et al. found mild to moderate dysfunction in 21% at long-term follow-up in FNP of various etiologies [10]. Skogman et al. found the same number (21%) of persistent FNP at the long-term follow-up of children with LNB [11], and in Peltomaa’s study on children’s acute FNP of varied etiologies, a mild to moderate dysfunction at follow-up was found in 23–29% of cases [12]. The persistent symptoms are described as excessive tear secretion, drooling or pronunciation problems, as well as cosmetic and social problems [11]. Synkinesis, defined as involuntary movement accompanying a voluntary one [3], was seen in 8% of children at the long-term follow-up in FNP associated with LNB [11]. Psychological distress and depression associated with FNP among children have been evaluated in a few studies. Lee et al. found a vast majority of children reporting distress associated with FNP at the 6-month follow-up [13], and a large retrospective cohort study revealed that children with a history of FNP were diagnosed with depression more often than matched controls [14].

Corticosteroid treatment is believed to reduce edema, inflammation and swelling of the facial nerve, thus leading to decompression of the nerve within the facial canal in the temporal bone [15]. Short-term corticosteroid treatment, initiated within 72 h after symptom onset, is widely recommended for treating idiopathic FNP in adults [16]. However, this has not always been the case, as the efficacy and safety of corticosteroids has long been subject to debate [3, 17]. Two large randomized controlled trials ultimately concluded that corticosteroid treatment significantly improves outcome in adult patients with idiopathic FNP [18, 19], and a subsequent Cochrane review has stated that further studies on the efficacy of corticosteroids on idiopathic FNP in adults are not needed [16]. Regarding children, a systematic review concludes that there are no high quality, well-powered studies to provide evidence of the efficacy and safety of corticosteroids in treating children’s acute FNP [1]. Articles published 2000–2010 were included in the review and the authors found no controlled trials, and level 4 publications predominated [1]. To our knowledge, no convincing evidence has been presented subsequent to this review.

Short-term, oral corticosteroid treatment is commonly used when treating children for a wide array of medical conditions and the safety has previously been evaluated in prospective trials [20]. The most commonly reported side effects among pediatric patients are vomiting, behavioral change and sleep disturbance, occurring in 4–5% of cases. Infections associated with treatment with short-course, oral corticosteroid appear in 1% of children. More serious infectious conditions, such as Varicella zoster, have been described in a few cases [20–22].

In summary, an acute FNP may cause a permanent impairment of the facial function, potentially with a life-long negative influence on a child’s quality of life, and the efficacy of corticosteroids has been shown to significantly improve clinical recovery in adults. Therefore, we are conducting a randomized double-blind, placebo-controlled multicenter trial in children, including pediatric patients with acute FNP of either idiopathic etiology or associated with LNB.

Our aim is to determine the efficacy of corticosteroid treatment in children with acute FNP in a *Borrelia burgdorferi* endemic area. We hypothesize that corticosteroid treatment favors complete recovery in children with FNP at the 12-month follow-up.

## Method/design

### Study design and setting

The FACE study (Facial nerve palsy And Cortisone Evaluation in children) is a double-blind, placebo-controlled, multicenter, superiority trial. Participants are randomized consecutively into two parallel groups with an allocation ratio of 1:1. The 12 study centers are pediatric departments in central-southern Sweden, all situated in *Borrelia burgdorferi* endemic areas. The study centers represent pediatric departments at large academic medical centers as well as at smaller secondary care centers. The full list of study centers is published at ClinicalTrial.gov (NCT03781700). Children are evaluated for LNB as part of the clinical routine work-up at each pediatric department, always including lumbar puncture with white cell count in cerebrospinal fluid and detection of intrathecally produced anti-*Borrelia* antibodies, in order to find and classify LNB cases [8]. The first participant in the FACE study was included May 3rd 2019 and recruitment is predicted to continue throughout 2022.

The FACE study is following the SPIRIT guidelines.

### Study population

Children 1–17 years of age presenting with an acute FNP within 72 h from onset are enrolled in this study. Exclusion criteria ensure exclusion of children with acute FNP associated with underlying medical conditions other than LNB. Thus, participants have a FNP of either idiopathic etiology or associated with LNB. Furthermore, children with conditions where corticosteroid treatment is inappropriate or unsafe are excluded (Table 1). All study participants are recorded in an identification log, containing information to enable identification of each participant in relation to a specific code number. Children with an acute FNP who are not eligible for the FACE study are recorded in a de-identified mode for gender, age-interval and reason for non-participation in the study.

**Table 1:** Inclusion and exclusion criteria

*Inclusion criteria*	
1. 1–17 years of age	
2. Acute peripheral unilateral facial nerve palsy	
3. Symptom duration < 72 h	
4. Signed informed consent	
*Exclusion criteria*	
1. Head trauma < 1 month	
2. Central facial palsy	
3. Malformations of the head and neck	
4. Conditions incompatible with corticosteroid treatment (arterial hypertension, diabetes mellitus type 1, psychiatric disorder, active or latent tuberculosis, intolerance of lactose)	
5. Current or past oncological diagnosis	
6. Other serious medical conditions (meningitis, encephalitis, stroke)	
7. Acute otitis media	
8. Signs of herpes simplex or varicella zoster infection (vesicles in the ear region)	
9. Pregnancy or breastfeeding	
10. Use of any systemic or inhaled corticosteroids within 2 weeks prior symptom onset	
11. Immunization with live vaccine < 1 month prior onset of symptoms	
12. Requirement of live vaccine within 2 months from start of experimental treatment (prednisolone/placebo)	
13. Evaluation of primary endpoint at 12 months not feasible for any reason	
14. Previously included into the FACE study	

### Intervention

The intervention group receives prednisolone (tablet Prednisolone 5 mg) for oral intake once daily for 10 days without taper. The dosage is set in weight intervals aiming at a dosage of 1 mg/kg/day (maximum 50 mg/day) (Table 2). The control group receives the corresponding amount of placebo tablets. Investigator and child/parents are blinded to the intervention. Thus, the prescription to the specific participant only includes the specific number of tablets of study drug, for oral intake once daily for 10 days. The study drug intervention and the protocol as a whole have been reviewed and approved by the Swedish medical products agency (EudraCT nr 2017–004187-35).

**Table 2:** Dosage of study drug (tablet Prednisolone 5 mg or placebo)

Child’s weight in kilograms	Number of tablets/day	Total number of tablets
5–9.9	2	20
10–14.9	3	30
15–19.9	4	40
20–24.9	5	50
25–29.9	6	60
30–34.9	7	70
35–39.9	8	80
40–44.9	9	90
≥45	10	100

Weigh intervals and number of tablets for oral intake once daily. Treatment period is 10 days. All bottles contain 100 tablets

The choices of corticosteroid and dosage are based on previous studies in adults and children. Engström et al. used prednisolone 60 mg/day for oral intake once daily for 5 days, and 5 days of taper in the Scandinavian Bell’s palsy study in adults [18]. Sullivan et al. used prednisolone 50 mg/day for oral intake once daily for 10 days without taper, in the corresponding study in adults in Scotland [19]. The dosage of 1 mg/kg/day (maximum 50 mg/day) is considered an equivalent dose in children and has been used in several small treatment studies in children, with or without taper [1, 23]. Thus, based on previous studies mentioned above, and to facilitate and uphold compliance with treatment, a dosage of 1 mg/kg/day (maximum 50 mg/day) in weight intervals (Table 2) without taper, was chosen for the FACE study. Furthermore, the chosen dosage strategy is in line with the treatment protocol of an ongoing trial on FNP in children in Australia and New Zeeland, using a 10-day course of prednisolone, aiming at 1 mg/kg/day for oral intake once daily without taper [24].

The tablets with the study drug can be crushed and mixed with something tasty in order to facilitate oral intake, with recognition from the Swedish medical product agency (MPA) Taking the first dose as soon as possible after inclusion and the decision about concomitant antibiotic treatment for LNB, is encouraged. Hence, a potential prednisolone treatment will not precede the start of antibiotics for those children with FNP associated to LNB. Taking subsequent doses in the morning is encouraged. Study nurses are provided with standardized recommendations for the participants in case of vomiting in conjunction with, or after intake of, the study drug. The bottle containing the study drug can be stored at room temperature.

#### Withdrawal of subjects

Participants can leave the study at any time without giving any specific explanation, but will be followed-up for safety reasons. Furthermore, the principal investigator can decide on withdrawal of a participant from the study due to e.g. adverse events or insufficient compliance with the study drug treatment.

#### Randomization and blinding

The study is double-blind with a concealed allocation. A study statistician was responsible for the randomization procedure. A randomization list, or code key, using variable random block sizes, was created and used when undertaking coding and labelling of bottles of the study drug (prednisolone/placebo) at the central pharmaceutical unit (Tamro AB). The coded bottles were distributed in advance to the study centers. On inclusion, each participant obtains a consecutive code number from the numbers present at the current study center, and receives a bottle of the study drug with the corresponding code number. The code key (identifying what code number corresponds to prednisolone or placebo) is kept sealed and secure at the Center for Clinical Research, Region Dalarna county, and at the central pharmaceutical unit.

#### Emergency un-blinding

There is a theoretical risk of situations occurring when urgent information on the actual treatment (prednisolone or placebo) is vital or essential. In case of such an emergency, the study center holds a sealed opaque envelope for each code, declaring the allocation (prednisolone or placebo) for the specific participant. To uphold the quality and the credibility of the trial, breaking the code in advance for a specific participant should only happen under extraordinary circumstances where it is necessary for the safety of the participant. Principal investigators will report such events to the coordinating investigator within 24 h of breaking the code.

### Concomitant therapy

Study participants who are classified as having a possible or confirmed LNB receive antibiotic treatment according to national guidelines, i.e. ceftriaxone IV 50–100 mg/kg (maximum 2 g) once daily for 10–14 days or doxycycline PO 4 mg/kg (maximum 200 mg) once daily for 10–14 days [8]. This, and other concomitant treatments, will be recorded in the clinical report form (CRF).

### Primary outcome

The primary outcome is complete recovery, defined as House-Brackmann grading scale (HBGS) grade I, at the 12-month follow-up. The HBGS is a clinical grading scale for describing the severity of facial impairment [25]. The physician grades the facial function on the affected side from I, meaning no impairment, to VI, corresponding to no function at all. The evaluation takes into account both static and dynamic asymmetry, as well as synkinesis. Previous studies on children have commonly used HBGS for evaluation of facial function, among them studies constituting the basis of our power calculations [10–12]. Hence, HBGS is preferred as the instrument for primary outcome measure in the FACE study. Furthermore, at the 12-month follow-up, the facial function has been shown to be stable and no further spontaneous improvement is expected [26].

### Secondary outcomes

Secondary outcomes include evaluation of complete recovery measured with the Sunnybrook facial grading system (SFGS), prediction of recovery with SFGS, evaluation of facial disability using three different instruments and monitoring of safety.

The SFGS is an alternative to HBGS, in which the physician evaluates each region of the face separately, in motion and at rest respectively, also accounting for synkinesis. The evaluation results in a composite score, where the maximum score 100 corresponds to full function in all regions of the face with no synkinesis [27]. In adults, the SFGS has been shown to have better repeatability and inter-rater agreement, as compared to HBGS [28]. Moreover, the SFGS has been shown to be reliable even with novice raters [28, 29], and this is yet another reason for including it in the protocol of the FACE study. The efficacy of prednisolone treatment will be evaluated using SFGS in addition to HBGS, defining complete recovery as SFGS composite score 100, at the 12-month follow-up. Furthermore, as a continuous variable SFGS will be useful for analyzing improvement as well as effect size. Hence, the SFGS composite score median value at 12 months constitutes yet another secondary outcome.

The possibility of prediction of complete recovery at the 12-month follow-up will be evaluated by analyzing the positive predictive value at different cut-off levels for the SFGS composite score at the 1-month follow-up. A previous study in adults has shown that prediction of an unfavorable outcome is possible at a 1-month follow-up using defined cut-off levels for the SFGS [30].

The FACE study will only include two follow-up visits. This is preferable, as we do not intend to analyze time to recovery as an outcome in the FACE study. By keeping the number of follow-ups to a minimum, we hope to improve attendance at the 12-month follow-up, which is the time point for our primary outcome measure.

The agreement between HBGS and SFGS as rating scales for facial function was merely moderate in a previous study of adults by Berg et al. [31]. Adding SFGS to the protocol will enable a corresponding agreement evaluation in a pediatric population.

Child/parent-perceived disability and quality of life are assessed with three different instruments as secondary outcomes, in order to describe the impact of FNP on a child’s wellbeing. Furthermore, these outcomes will be evaluated for correlation to physician-assessed grading by HBGS and SFGS at the 1- and 12-month follow-ups. Two disease-specific quality of life questionnaires and one questionnaire evaluating presence of synkinesis are used, namely the Facial Clinimetric Evaluation (FaCE) scale, the Facial Disability Index (FDI) and the Synkinesis Assessment Questionnaire (SAQ) [32–34]. These instruments are child/parent-assessed questionnaires, originally developed for an adult population but also now linguistically adapted to better suit a pediatric population. Each questionnaire allows answers from the child or parent, alternatively from the child with assistance from the parent. These modified questionnaires have been evaluated using a “think-aloud” interview method [35, 36] in a few children, with present or previous FNP, showing satisfactory usability (unpublished data).

Additionally, the number of adverse events are assessed as a secondary outcome comparing treatment/placebo groups in order to evaluate the safety profile of prednisolone.

All primary and secondary outcomes will be evaluated in all patients and compared in the treatment/placebo groups as well as in subgroup analysis comparing children with idiopathic FNP and children with FNP associated to LNB.

### Study procedure

#### At inclusion/baseline

Children presenting with an acute FNP at any of the 12 study centers are screened for eligibility for the study. This can take place either at the emergency department or at the pediatric department. If eligibility criteria are met and the child and parents choose to participate, information about the study procedure is given to the family and an informed consent is signed by the parents. In accordance with Swedish legislation, both parents must approve of the child’s participation in a trial concerning drug evaluation, by written consent. At inclusion, the participant receives a consecutive code number and the corresponding bottle of study drug (prednisolone/placebo). Baseline data including demographics, medical history, concomitant medication and relevant findings from the physical examination are collected by the investigator (pediatrician). The neuroborreliosis prediction (NeBoP) score [37] will be recorded, as part of the clinical routine, for a decision about antibiotic treatment.

Furthermore, facial function is graded at inclusion, using the HBGS and the SFGS, by the investigator (either a pediatrician or an otorhinolaryngologist). Children with suspected LNB associated FNP receive antibiotic treatment as part of the routine work-up at each study center.

#### At 1 and 2 weeks post-inclusion

At 1 and 2 weeks post-inclusion, the child/parent is contacted by telephone by a study nurse, to verify and enhance compliance with the study drug treatment and to check for adverse events. In the case of adverse events, they are documented and evaluated as described below. No specific questions on the facial function are addressed at this time.

#### At 1 and 12 months post-inclusion

Follow-up visits to the investigator (either a pediatrician or an otorhinolaryngologist) take place at 1 and 12 months post-inclusion. At the 1-month follow-up visit, the facial function is evaluated using the physician-assessed HBGS and the SFGS. The child and/or parent complete the FaCE scale and the FDI questionnaires. The bottle of study drug is collected and controlled for remaining number of tablets for each specific participant. The importance of attending the upcoming 12-month follow-up visit is stressed for the child and parent.

At the 12-month follow-up visit, the facial function is evaluated using the physician-assessed HBGS and the SFGS. The child and/or parent completes the FaCE scale, the FDI and the SAQ questionnaires. If a child is in need of additional follow-up visits regarding the facial function during the study period, this can be offered independently of the study schedule, but is recorded in the CRF at the 12-month visit, as is any additional specific treatment targeting the facial function (e.g. physiotherapy, acupuncture etc.).

The time schedule for the FACE study is shown in Table 3.

**Table 3:** Study schedule

	Baseline/Inclusion	Telephone call 1 week ± 3 days	Telephone call 2 weeks ± 3 days	Visit 1 month ± 5 days	Visit 12 months ± 2 weeks
Written informed consent	X				
Inclusion /Exclusion criteria	X				
Allocation of code number	X				
Medical history	X				
Demographics	X				
Weight/length/BMI	X				
Physical examination	X				
NeBoP score	X				
House-Brackmann grading scale (HBGS)	X			X	X
Sunnybrook grading system (SFGS)	X			X	X
Concomitant medication	X			X	X
Initiation of study treatment	X				
Compliance with study drug		X	X		
Reporting of Adverse Events (AE)		X	X	X	X
Return of bottles of study drug				X	
Facial disability index (FDI)				X	X
Facial Clinimetric Evaluation (FaCE) Scale				X	X
Synkinesis Assessment Questionnaire (SAQ)					X

### Sample size estimation and recruitment

In the FACE study, the sample size is calculated on the primary outcome (i.e. complete recovery, defined as HBGS grade I, at the 12-month follow-up visit). Previous studies have shown spontaneous complete recovery (HBGS = I) to occur in approximately 80% of untreated children with an acute FNP [10–12], this is the estimated percentage for complete recovery in the control group. Furthermore, in accordance with previous studies in adults [18], we consider a clinically important difference between groups to be 10%, hypothesizing the intervention to cause an increase in complete recovery rate from 80 to 90% in the treatment group. To be able to show a 10% difference between groups with statistical significance (*p* < 0.05) and a power of 80% [38], a total of 500 participants (250 in each group), is needed for the trial, allowing 10% of participants to be lost to follow-up.

In order to promote progress and ensure high quality of study procedures, investigators and study nurses received education in Good Clinical Practice before the study start and attend yearly meetings available to all personnel. Investigators are thoroughly instructed in applying the HBGS and the SFGS for reliable evaluation of facial impairment, and at the 1- and 12-month follow-ups, only one or two investigators per study center will apply the HBGS and the SFGS. Recruitment rate is enhanced by continuous contact between the coordinating investigator, the principal investigators and study nurses at each study center, throughout the study period.

### Evaluation of harms

At all contacts with the child/parent (telephone or visits), any testimonial of adverse events (AE) or examination findings suggesting an AE, is noted and evaluated regarding intensity and causality. An AE is any unfavorable, unintended clinical sign, symptom or medical complaint during the study period. Serious adverse events (SAEs) are defined as any AE that results in death, is life threatening, requires inpatient hospitalization, requires prolongation of existing hospitalization, results in persistent or significant disability/incapacity or is a congenital anomaly/birth defect. All SAEs are to be reported to the sponsor whether or not considered as related to the study drug. Pre-planned hospitalization for a pre-existing condition that did not worsen during the course of the study period is not considered as an SAE.

The Swedish medical products agency (MPA) receives annual reports from the FACE study regarding safety issues including SAEs. In the rare event of a suspected unexpected serious adverse reaction (SUSAR), the sponsor reports this to the Swedish MPA for documentation in the EudraVigilance database, as soon as possible. A SUSAR is defined as an SAE with a reasonable likelihood of a causal relationship to the study drug.

A Data Safety Monitoring Board (DSMB) evaluates safety data regarding AEs and SAEs at predetermined intervals. For the members in the DSMB, the working procedures and duties are clearly defined.

#### Insurance

Participants in this study are covered by the Swedish Patient Insurance and the Swedish Pharmaceutical Insurance.

### Statistics and data management

#### Data collection

All data in the FACE study, including questionnaires completed by the children/parents (FaCE scale, FDI, SAQ), are recorded as paper CRFs for each participant. The coordinating investigator is responsible for collecting and entering data from the paper CRFs into a database (managed by the Swedish data management company MediCaseAB). The original and a copy of the paper CRFs will be archived by the sponsor, and the study center respectively, for at least 10 years. An original copy of the CRFs (including questionnaires) can be made available upon request to the corresponding author.

### Statistical analysis

A study statistician will be responsible for the statistical evaluation of results from the study after study closure. A study-specific statistical analysis plan (SAP) will be prepared by the study statistician, where further details will be specified. No interim analyses will be performed, and no data will be given on the personal level, only presented in subgroups or at group level.

Intention to treat will be applied when analyzing results from the primary outcome. In the primary analysis of treatment efficacy, the difference between the intervention group and the control group will be determined, regarding the proportion of children classified as having complete recovery (HBGS I) at the 12-month follow-up. A Chi^2^ test will be performed with risk estimate and 95% confidence interval. In addition, treatment efficacy will be evaluated with a Chi^2^ test regarding our second outcome measure, complete recovery defined as SFGS composite score 100. Furthermore, efficacy will be evaluated by using SFGS as a continuous variable, comparing composite score medians between the intervention group and control group (Mann-Whitney *U*-test) and for calculation of effect size with Cohen’s d.

For correlation between scales with continuous properties, the Spearman’s test will be used, whereas for scales with categorical properties, agreement with Cohen’s kappa will be applied. When predicting complete recovery at the 12-month follow-up based on grading at the 1-month follow-up, diagnostic performance will be calculated on complete recovery defined as HBGS I and SFGS composite score 100. Logistic regression analysis will be performed to evaluate complete recovery at the 12-month follow-up in relation to baseline data and treatment, as well as when performing subgroup analysis.

### Quality control

The sponsor provides monitoring of the study. The data monitoring committee (DMC) is strictly independent from the sponsor and does not have other competing interests. The DMC periodically monitors the study centers in order to ensure compliance with the protocol, principles of Good Clinical Practice and applicable regulatory requirements. Source documents are reviewed for verification of consistency with the data recorded in the paper CRFs. The DMC also provides information and support to the principal investigators and study nurses at each study center. In addition, a quality assurance audit by the sponsor or by the Swedish MPA, may be performed at any study center.

### Ethical considerations

This study is conducted in accordance with applicable regulatory requirements, the principles of Good Clinical Practice and the ethical principles of the declaration of Helsinki, as adopted by the 18th World Medical Assembly in Helsinki, Finland, in 1964, and subsequent versions. A risk-benefit analysis was made when developing this protocol. The Regional Ethical Review Board in Uppsala, Sweden approved the clinical study protocol, including forms for informed consent and the participant information form (Dnr 2017/554 and 2019–01546).

All potential changes to the final study protocol will be documented by signed protocol amendments. If substantial changes to the design of the study are made, the MPA and the Ethical Review Board will be notified for review and renewed approval.

#### Consent to participate

The process of acquiring informed consent is undertaken by the principal investigator at each center or by another investigator delegated by him/her. The child is informed at a level of his/her understanding, and the child’s integrity and autonomy are to be respected, although the consent form only requires to be signed by parents.

#### Confidentiality

All data are recorded in de-identified (coded) paper CRFs, stored in locked rooms at each specific study center. The Subject Enrolment and Identification log linking a specific participant to a specific code is to be kept secure and separate from CRFs at each study center. The study database will be secured by password for entering data during the study period and for analyzing data after the closure of the database. Data will be available to the research group only.

#### Dissemination policy

After completion of the study, the Sponsor, in cooperation with the principal investigators, will present a clinical study report to the MPA. Results will be published in peer-reviewed scientific journals. Professional medical writers will not be used. The clinical study protocol of the FACE study is published and can be read in full at Clinicaltrials.gov (NCT03781700).

## Discussion

Acute FNP in children poses a clinical challenge due to its etiological heterogeneity and uncertain treatment strategies. Up to now, it is unknown whether children with acute FNP benefit from corticosteroid treatment to the same extent that has previously been shown in idiopathic FNP in adults. The off-label prescription of drugs to pediatric patients is extensive [39, 40]. This has been problematized increasingly in recent years and legislative efforts have been taken within the EU and USA to promote the inclusion of children in clinical trials, regarding both new and established drugs [41–44]. The FACE study, determining the efficacy of prednisolone in children with acute FNP, can be conducted without ethical difficulties and with little risk of harm. Therefore, it is our firm opinion that evidence from one or several such trials should precede recommendations for the use of corticosteroid treatment in this group of patients.

Furthermore, the large proportion of acute FNP associated with Lyme neuroborreliosis (LNB) in children, calls for determination of efficacy of corticosteroid treatment in this group also, which has not previously been thoroughly studied in children or in adults. Evaluation of potentially harmful effects of corticosteroid treatment is another aspect of great interest, particularly in the LNB group. This aspect of potentially harmful effects has been emphasized in two recent publications [45, 46]. Jowett et al. present results indicating a less favorable outcome in patients with FNP associated with LNB being treated with corticosteroids in addition to antibiotics, as compared to patients being treated with antibiotics alone [45]. The study has limitations in being retrospective, having an undefined follow-up period and a high risk of patients’ self-selection bias. Furthermore, the mean age was 39.6 years (range 6–72) among included patients and only a few cases were pediatric patients. Wormser et al. prospectively followed a small number of adult FNP cases (*n* = 11) being treated with corticosteroids in addition to antibiotics, and found an unfavorable outcome (self-reported persistent facial weakness yes/no) in 55% of cases at follow-up [46]. The study had no control group, a binary evaluation method, a high risk of referral bias and the definition of the LNB diagnosis was not declared. Given the limitations of the two publications, the ultimate contributions of these studies are that they illustrate that prospective randomized clinical trials are warranted, which is also righteously stressed by the authors. This further supports the strong intention of finding evidence-based treatment guidelines, to include children with acute FNP associated with LNB.

As far as we know, this protocol outlines the first pediatric trial on the efficacy of corticosteroid treatment in acute FNP, including both children presenting with FNP of idiopathic etiology and FNP associated with LNB. A protocol article published in 2017 reveals an ongoing study in New Zealand and Australia, the BellPIC study, with the aim of evaluating the efficacy of corticosteroid treatment in children with idiopathic FNP [24]. Thus, the BellPIC study does not include children with FNP associated with LNB, and the FACE study will constitute a complement to the BellPIC study with a similar approach but in a different setting. Other differences include the primary outcome where the BellPIC study uses complete recovery (defined as HBGS I) at the 1-month follow-up, as compared to the 12-month follow-up in the FACE study. Furthermore, the intervention, being similar in dose and drug of choice (prednisolone 1 mg/kg/day for 10 days), differs however in the preparation of the drug, as BellPIC uses oral liquid as compared to oral tablets in the FACE study. Nevertheless, the two studies are comparable in many respects and may be included in future meta-analyses.

Taking into consideration previous research on children’s facial palsy referred to in this article, and the clinical experiences of the authors, the HBGS seems to be the most widely used grading scale for evaluating children’s FNP. In adult patients, the SFGS is recommended rather than the HBGS in several articles [28, 47]. We expect the FACE study to shed light on the usability of the SFGS in pediatric patients. However, physician-assessed evaluation of facial impairment alone might not reveal the whole truth regarding persisting symptoms following children’s FNP. One study investigating long-term outcome in children with a history of FNP associated with LNB, found no correlation between physician-assessed grading (HBGS) of facial impairment and subjective feeling of facial disability [48]. Even though small in size (24 participants), this study illuminates the complexity of evaluating persistent signs and symptoms following FNP, and strongly suggests that instruments for subjective facial disability should be included as patient reported outcome measures (PROMs) in a trial such as the FACE study.

The Facial nerve palsy And Cortisone Evaluation (FACE) study in children, now with ongoing recruitment, will contribute a large amount of data enabling evaluation of disease-specific quality of life, clinical grading instruments and prediction of recovery, in addition to determining the efficacy of corticosteroids in children’s facial nerve palsy. The results will potentially establish the foundation for new national and international evidence-based guidelines on how to treat children with acute FNP, applicable also in *Borrelia burgdorferi* endemic areas.

## Data Availability

Not applicable.

## Abbreviations

AE: Adverse event
CRF: Clinical report form
DMC: Data monitoring committee
DSMB: Data safety monitoring board
FaCE scale: Facial clinimetric evaluation scale
FACE study: Facial nerve palsy And Cortisone Evaluation study
FDI: Facial disability index
FNP: Facial nerve palsy
HBGS: House-Brackmann grading scale
LNB: Lyme neuroborreliosis
MPA: Medical product agency
NeBoP score: Neuroborreliosis prediction score
PROM: Patient reported outcome measure
SAE: Serious adverse event
SAP: Statistical analysis plan
SAQ: Synkinesis assessment questionnaire
SFGS: Sunnybrook facial grading system
SUSAR: Suspected unexpected serious adverse reaction
